# PVP as an Oxygen Vacancy-Inducing Agent in the Development of Black 45S5 Bioactive Glass Fibrous Scaffolds Doped with Zn and Mg Using A-HSBS

**DOI:** 10.3390/ma18061340

**Published:** 2025-03-18

**Authors:** Keila C. Costa, Maria Geórgia da S. Andrade, Rondinele N. de Araujo, Adegildo R. de Abreu Junior, Marianna V. Sobral, Juan Carlos R. Gonçalves, Bianca V. Sousa, Gelmires A. Neves, Romualdo R. Menezes

**Affiliations:** 1 Graduate Program in Materials Science and Engineering (PPG-CEMat), Federal University of Campina Grande (UFCG), Av. Aprígio Veloso-882, Bodocongó, Campina Grande 58429-900, PB, Brazil; maria.georgia@estudante.ufcg.edu.br; 2 Laboratory of Materials Technology, Department of Materials Engineering, Federal University of Campina Grande (UFCG), Av. Aprígio Veloso-882, Bodocongó, Campina Grande 58429-900, PB, Brazil; rondinele.nunes@estudante.ufcg.edu.br (R.N.d.A.); gelmires.neves@ufcg.edu.br (G.A.N.); 3 Graduate Program in Natural and Synthetic Bioactive Products, Onco Pharmacology Laboratory, Federal University of Paraíba (UFPB), Campus I, Castelo Branco, s/n, University City, João Pessoa 58051-970, PB, Brazil; abreu.jr.18@gmail.com (A.R.d.A.J.); mariannavbs@gmail.com (M.V.S.); juan.goncalves@academico.ufpb.br (J.C.R.G.); 4 Department of Chemical Engineering, Federal University of Campina Grande (UFCG), Av. Aprígio Veloso-882, Bodocongó, Campina Grande 58429-900, PB, Brazil; bianca.viana@ufcg.edu.br

**Keywords:** black glasses, fibrous scaffolds, air-heated solution blow spinning, inorganic ions

## Abstract

Currently, there is an increasing demand for advanced materials that can address the needs of tissue engineering and have the potential for use in treatments targeting tumor cells, such as black bioactive materials in photothermal therapy. Thus, 3D fibrous scaffolds of black 45S5 bioactive glass were produced using the air-heated solution blow spinning (A-HSBS) technique, with polyvinylpyrrolidone (PVP) serving as a spinning aid and an oxygen vacancy-inducing agent. Glass powder with the same composition was synthesized via the sol-gel route for comparison. The samples were characterized using thermogravimetric analysis, X-ray diffraction, FTIR spectroscopy, and scanning electron microscopy, along with in vitro tests using simulated body fluid (SBF), phosphate-buffered saline (PBS), and TRIS solution. The results showed that PVP enhanced oxygen vacancy formation and stabilized the scaffolds at 600 °C. Doping with Zn and Mg ions reduced crystallization while significantly increasing the fiber diameters. Scaffolds doped with Zn exhibited lower degradation rates, delayed apatite formation, and hindered ionic release. Conversely, Mg ions facilitated greater interaction with the medium and rapid apatite formation, completely covering the fibers. The scaffolds showed no cytotoxicity in the MTT assay at concentrations of up to 200 µg/mL for HaCat cells and 0.8 mg/mL for L929 cells. This study demonstrated the effectiveness of using PVP in the production of black bioactive glass scaffolds, highlighting their potential for bone regeneration.

## 1. Introduction

The 45S5 glass (Bioglass^®^), developed by Larry L. Hench and colleagues, was the first effective material for bone tissue repair [1] and is commercially available from Mo-Sci Corporation, Rolla, MO, USA. Its composition includes SiO_2_ (45% by weight), Na_2_O (24.5% by weight), CaO (24.5% by weight), and P_2_O_5_ (6% by weight) [1,2]. This bioactive glass (BG) is commonly used in tissue regeneration due to its excellent physiological dissolution behavior in body fluids, as it is biocompatible and bioabsorbable, exhibiting osteoinductive and osteogenic properties [3,4,5].

On the other hand, due to the difficulty of effectively targeting or treating cancer cells directly, few studies have explored the structure of the commonly synthesized 45S5 glass for bone cancer treatment [6]. Alternative approaches, such as the transport of antitumor drugs [7] and hyperthermia, have been employed [8,9,10,11,12]. However, challenges already exist, and new therapeutic strategies are needed as complementary and beneficial alternatives, allowing for the elimination of tumor cells while promoting the healthy recovery of surrounding tissues, thereby improving treatment efficacy and minimizing collateral damage during the healing process [13].

Black bioceramics have emerged as promising multifunctional materials due to their high bioactivity and ability to regenerate tissues, as well as their effectiveness in photothermal and photodynamic applications for non-invasive cancer therapies [14,15,16,17]. They can convert near-infrared (NIR-II, 1000–1400 nm) light energy into heat or reactive oxygen species, effectively destroying cancer cells during these therapies. These properties make these bioceramics useful in cancer treatment, where their antitumor potential contributes to tissue repair and enhances bioactivity [18]. The black coloration of these materials results from microstructural changes caused by oxygen vacancies [19,20,21].

Black Si–Ca–P-based glass-ceramic nanoparticles (BBGN), containing molybdenum nanocrystals, have been synthesized [22] for multifunctional applications. These nanoparticles have demonstrated excellent photometric and antioxidant activity, as well as antibacterial properties, effectively inhibiting tumor recurrence and promoting wound healing. These effects are attributed to the presence of free electrons and abundant oxygen vacancies in the structure of the bioactive glass.

Inorganic materials, such as black titania nanoparticles (B-TiO_2_-x), have been synthesized through reduction with Mg and incorporated into a chitosan matrix. Due to the high presence of oxygen vacancies, these nanoparticles facilitated the simultaneous combination of photothermal therapy (PTT) and photodynamic therapy (PDT) under near-infrared laser irradiation. This system resulted in excellent therapeutic effects in the treatment of skin tumors, both in vitro and in vivo [23].

On the other hand, nanofibrous scaffolds serve as temporary molds that mimic the extracellular matrix, allowing for better adhesion and proliferation of surrounding living tissue cells [24,25,26]. The solution blow spinning (SBS) technique has been demonstrated to be efficient in producing scaffolds of bioactive materials [27]. However, the processing of low volatile solvents is still a challenge, and the air-heated solution blow spinning (A-HSBS) method was proposed by Silva [28], adapting the SBS. A-HSBS presented greater efficiency in producing biphasic calcium phosphate (BCP) [28] and ICIE16 bioactive glass (ICIE16-BG) [29] nanofibrous scaffolds. In the cited studies, PVP was used as a spinning aid, promoting the formation of continuous fibers, and improving the structural stability of the scaffolds. However, despite the studies on the synthesis of 45S5 bioactive glass nanofibers through electrospinning [30,31,32], no research was observed on the production of pure or doped 45S5 fibers by SBS or A-HSBS.

Considering the interplay of factors to enhance the properties of bioactive glasses (BGs), the controlled release of therapeutic ions stimulates protein and cell attachment and promotes cell proliferation [11]. Among these ions, zinc (Zn) is used in bioactive glasses due to its beneficial effects on tissue regeneration, such as stimulating protein synthesis and inhibiting bone resorption, along with its high antimicrobial potential. Magnesium (Mg) also plays a crucial role in bone mineralization. Studies have shown that doping with magnesium stimulates osteogenesis, both in vitro and in vivo, without cytotoxic effects [33,34]. Mg positively influences the activities of osteoblasts and osteoclasts [35,36] and improves cell viability in bioactive glasses [37]. Additionally, the incorporation of Mg and Zn ions, even at low concentrations, can enhance the processability of 45S5 glass powders and slow down the crystallization process [38,39].

Therefore, the objective of this work was to produce black nanofibrous scaffolds of 45S5 bioactive glass using PVP as a spinning aid, doped with Zn and Mg at different concentrations, and to evaluate in vitro their influence on biomineralization, biodegradable and cytotoxic behavior, as well as on ionic release and cytotoxicity. The air-heated solution blow spinning (A-HSBS) technique was used for the production of 3D fibrous glasses, and, additionally, 45S5 glass powder was produced via the sol-gel route to compare the in vitro bioactivity and biodegradability.

## 2. Materials and Methods

### 2.1. Materials

The inorganic ion precursors used in this work were tetraethyl orthosilicate (TEOS) Si(OC_2_H_5_)_4_ (Sigma Aldrich, 99%, St. Louis, MO, USA), triethyl phosphate (C_2_H_5_O)_3_PO (Sigma Aldrich, ≥99.8%, St. Louis, MO, USA), calcium nitrate tetrahydrate Ca(NO_3_)_2_•4H_2_O (Sigma Aldrich, ≥99%, St. Louis, MO, USA), sodium nitrate NaNO_3_ (Isofar, ≥98%, Duque de Caxias, RJ, Brazil.), zinc nitrate hexahydrate Zn(NO_3_)_2_•6H_2_O (Sigma Aldrich, ≥98%, St. Louis, MO, USA), and magnesium nitrate hexahydrate Mg(NO_3_)_2_•6H_2_O (Neon, ≥98%, Suzano, SP, Brazil). Additionally, acetic acid CH_3_COOH (Neon, ≥99.8%, Suzano, SP, Brazil) and ethanol (Neon, ≥99.5%, Suzano, SP, Brazil), were used in the synthesis process. Polyvinylpyrrolidone (PVP) (Sigma Aldrich, average molecular weight ~1,300,000, St. Louis, MO, USA) was used as a spinning aid.

### 2.2. Methods

To produce 3D fibrous scaffolds (BG-F), first, the PVP was completely dissolved in 10% (*w*/*v*) ethanol under constant stirring at room temperature. The inorganic precursors were dissolved separately in distilled water and stirred for 60 min. Then, all the precursor solutions were added slowly dropwise in the PVP solution, followed by an additional 12 h of stirring. To catalyze the hydrolysis of TEOS, an acetic acid solution at a concentration of 1 M was added in a ratio of 1:1 (*v*/*v*) in the TEOS solution. The final solution was used in the spinning process. The compositions of the BGs used in this work are presented in Table 1.

The hybrid fibers (PVP + ceramic precursors) were then produced by A-HSBS. The arrangement used was detailed in Silva’s methodology [28]. The A-HSBS apparatus consists basically of an SBS setup (compressed air source, injection pump, spinning matrix, and a collector) and heat guns to aid solvent evaporation. The injection rate used was 6.6 mL/h, with a working distance of 400 mm, a protrusion of 10 mm, and a pressure of 25 psi. The temperature in the collector was maintained at 50 °C, with humidity below 30%. The fibers deposited on the collector formed a 3D hybrid scaffold. After spinning, the scaffolds were subjected to calcination at 300 °C for one hour, followed by a subsequent calcination at 600 °C for one hour (EDG, 3000), with a heating rate of 1 °C/min.

In the production of glass powders (BG-P) by the sol-gel route (Table 1), TEOS, TEP, and calcium nitrate were each dissolved separately in ethanol, while sodium nitrate was dissolved in distilled water. All solutions were stirred at room temperature until complete dissolution was achieved. Glacial acetic acid was added in TEOS solution in a ratio of 1:1.5 (*v*/*v*) of TEOS to catalyze the alcoholysis process. The other precursor solutions were then added dropwise to the TEOS solution. After the addition of the precursors, the final solution was heated to 70 °C for 12 h under stirring to promote gel formation. Then, the gel was dried in an oven at 150 °C for 24 h. The 45S5 glass powder was obtained by grinding the dried gel and its calcination at 800 °C for 3 h, with a heating rate of 10 °C/min.

### 2.3. Characterizations

The thermogravimetric analysis (TGA) of the dry gel and the hybrid scaffold (PVP-BG) was performed in an air atmosphere, with a heating rate of 5 °C/min, from room temperature to 1000 °C (Shimadzu, DTG-60H, Kyoto, Japan). X-ray diffraction was conducted using CuKα radiation (40 kV/30 mA) in fixed time mode, covering the range 2θ of 5° to 70° (Shimadzu, XRD-6000, Kyoto, Japan). Peak identification was performed using the Malvern Panalytical HighScore Plus software (Version 3.0e, 3.0.5, Almelo, The Netherlands, 2012).

The morphology of the scaffolds and powder was examined by scanning electron microscopy (SEM, VEGA 4, TESCAN, Brno, Czech Republic), and the diameters of at least 100 random fibers/particles were measured using ImageJ software (National Institutes of Health, USA, version 1.53t, Bethesda, MD, USA, 2021). The results were expressed as mean ± S.E.M. and analyzed by ANOVA followed by the Tukey test, considering significance at *p* < 0.05.

Fourier-transform infrared spectroscopy (FTIR) was performed with a PerkinElmer spectrometer (PerkinElmer, Waltham, MA, USA), with a resolution of 4 cm^−1^, 64 scans, and analyzed the absorption region between 4000 and 400 cm^−1^.

The porosity of the scaffolds was determined using a method based on Archimedes’ principle [40]. The samples were prepared in triplicate and immersed in high-purity ethanol for 2 h. Ethanol was chosen due to the high solubility of the scaffolds in water, which could compromise the accuracy of the results. Measurements were taken under three conditions: dry weight (*Wd*), submerged weight (*Wsub*), and wet weight *(Wwet*). The density of ethanol used in the calculations was ρe = 0.791 g/cm^3^.

Based on these measurements, the pore volume (*Vp*) and the solid structure volume (*Vs*) were determined using the following Equations (1) and (2):

(1) $$Vp=\frac{Wwet-Wsub}{\rho e}$$

(2) $$Vs=\frac{Wd-Wsub}{\rho e}$$

The porosity (e) was then calculated as the ratio between the pore volume and the total scaffold volume using Equation (3).

(3) $$e=\frac{Vp}{Vp+Vs}=\frac{Wwet-Wsub}{\left(Wwet-Wsub\right)+(Wd-Wsub)}\times 100\%$$

The in vitro biomineralization test was conducted using simulated body fluid (SBF) following the methodology of Kokubo and Takadama [41]. The solution in contact with the samples was replaced every 72 h, and the pH was measured regularly. For analysis, 15 mg of the samples were immersed in 20 mL of SBF at intervals of 24, 72, 120, and 168 h. All the compositions studied were analyzed in duplicate.

The surface roughness of the scaffolds was analyzed using field emission scanning electron microscopy (FEG-SEM, Mira 4, TESCAN, Brno, Czech Republic) with a profile line analysis system at a magnification of 200 kx. Images were acquired from different representative regions of the surface, and the analysis was conducted along a 1-micrometer (1000 nm) line. Roughness parameters were determined from the intensity profile graphs, considering Ra (arithmetic mean roughness), Rq (root mean square roughness), and Rz (maximum peak-to-valley height). Measurements were performed before and after scaffold immersion in simulated body fluid (SBF) for 7 days.

To evaluate the in vitro degradation, phosphate-buffered saline (PBS) was used. Samples with a concentration of 6 mg/mL were immersed in the PBS solution with a pH of 7.4 and a concentration of 0.01 M at intervals of 1, 3, 7, and 14 days, and all tests were performed in duplicate. The pH of the solution was measured after each immersion period. Subsequently, the samples were washed with distilled water and dried. The degradation (%) was evaluated using the initial weight (*Wo*) and the dry weight after immersion (*Wf*), according to Equation (4) [42]:

(4) $$\mathrm{Degradation}\;(\%)=\frac{\left(Wf-Wo\right)}{Wo}\times 100\%$$

The ionic release from the scaffolds was evaluated by immersing the samples in TRIS (tris(hydroxymethyl)aminomethane) solution, with the pH adjusted to 7.4, maintained at a temperature of 36.5 °C, over periods of 1, 3, 7, and 14 days. After each interval, the samples were filtered, and the collected solutions were analyzed using a Microwave Plasma Atomic Emission Spectrometer (Agilent Technologies, MP-AES 4200, Santa Clara, CA, USA). This test was performed in triplicate.

The MTT reduction colorimetric assay (3-(4,5-dimethylthiazol-2-yl)-2,5-diphenyl tetrazolium bromide) was used to evaluate the cytotoxicity of substances in an immortalized human keratinocyte cell line (HaCaT). HaCaT cells were seeded at a density of 3 × 10^5^ cells/mL in 96-well plates and incubated for 24 h at 37 °C in a 5% CO_2_ atmosphere. The cells were then treated with different concentrations of the substances (25–200 µg/mL) for 72 h. Plate readings were performed using a microplate spectrophotometer (Synergy HT, BioTek, Winooski, VT, USA) at an absorbance of 570 nm. The results were expressed as the mean ± standard error of the mean (S.E.M.) and analyzed using one-way Analysis of Variance (ANOVA), followed by the Tukey test. The experiments were performed in triplicate, and results were considered significant when *p* < 0.05. The assay was conducted with HaCaT keratinocytes, derived from human foreskin, acquired from the Cell Bank of Rio de Janeiro (BCRJ), at the OncoPharmacology Laboratory (João Pessoa, Brazil), linked to the Postgraduate Program in Natural and Synthetic Bioactive Products.

The MTT test was also performed to evaluate in vitro cytotoxicity in L-929 fibroblastic cells, following the criteria established by ISO 10993-5: 2009 [43] for cell viability (ASTM, 2011). For the preparation of extracts, 0.8 mg of each sample was previously sterilized by UV radiation and transferred to sterile vials containing 1 mL of PBS. The samples were incubated at 37 °C under continuous agitation for 24 h. L-929 cells were cultured in 96-well plates for 24 h. After cultivation, 50 µL of the filtered extracts were added to the respective wells and incubated again for 24 h. At the end of the incubation period, absorbance was measured using a microplate spectrophotometer (Victor X3, PerkinElmer, Waltham, MA, USA) at 570 nm, with a reference filter set to 650 nm. The assay was conducted with L-929 fibroblastic cells, derived from mouse fibroblasts (L929, clone 29, from Wistar rats), acquired from the Cell Bank of Rio de Janeiro (BCRJ), at the CERTBIO laboratory (Campina Grande, Brazil), accredited by ABNT ISO/IEC 17025:2005 [44], CRL 0799 for Chemical and Biological Testing.

## 3. Results and Discussion

**Figure 1:**
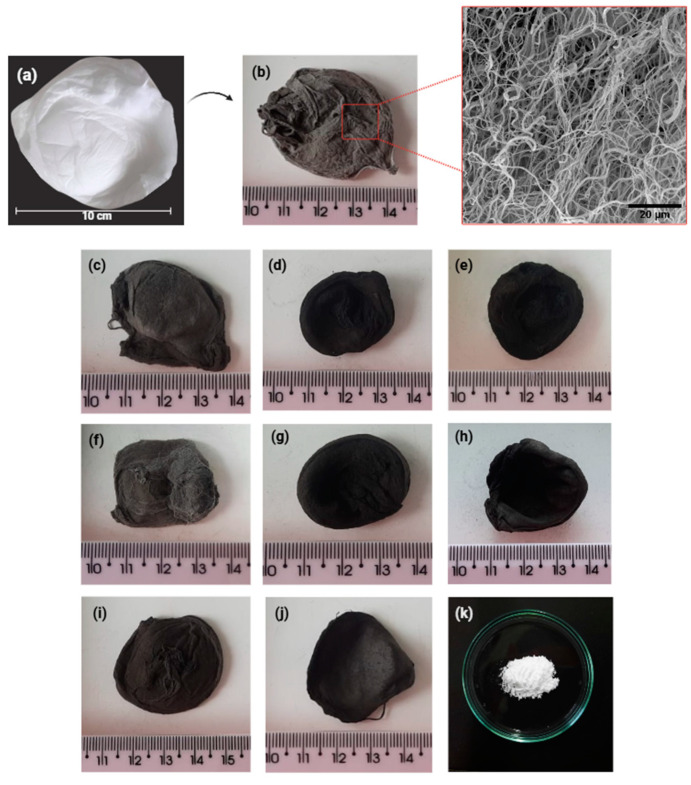
Photograph of fibrous scaffolds before (**a**) and after calcination/stabilization: (**b**) BG-F, (**c**) BG-Zn1, (**d**) BG-Zn3, (**e**) BG-Zn5, (**f**) BG-Mg1, (**g**) BG-Mg3, (**h**) BG-Mg5, (**i**) BG-Zn/Mg1, (**j**) BG-Zn/Mg3, and (**k**) BG-P.

Figure 1 presents the pure fibrous scaffold before and after calcination/stabilization. All doped fibrous samples exhibited a similar visual appearance. After the spinning process, the BG-PVP fibrous scaffolds displayed a three-dimensional structure with a white color, resembling cotton wool (Figure 1a). However, following calcination, the pure sample took on a gray hue (Figure 1b), while the doped samples turned black. All samples maintained their three-dimensional structure but became semi-flexible after calcination. Similar 3D structures of bioactive glasses produced by air-assisted spinning methods have been reported only in studies on 63S and 58S [45] using SBS (but composition without sodium) and in a study on ICIE-16 [29] using A-HSBS3.

The black coloration of the scaffolds may be associated with the presence of oxygen vacancies in the structure of the glasses. During the synthesis of the fibers, PVP, used as a spinning aid, acts as a matrix that traps metal ions through electrostatic interactions [46]. As the solvent evaporates and the fibers form, the ions become immobilized within the polymer chains [47,48]. At this stage, both metallic cations and anions (oxygen atoms) may be present in the matrix; however, cations tend to bond more strongly to the matrix than anions due to ionic-dipole interactions with the amine group [49,50].

The calcination was carried out in a static air atmosphere. Thus, the thermal decomposition of PVP can cause carbon to react with oxygen atoms, promoting reduction and releasing gases, such as CO and CO_2_. This process leads to a rearrangement of the glass structure [51]. Under high-temperature conditions, the oxygen atoms present, as well as those initially bonded to the structure, may be removed, and released as O_2_ gas. This removal of oxygen atoms results in the creation of “holes” in the vitreous network, forming vacancies [52,53,54]. Furthermore, the rearrangement of ions may lead to the formation of crystalline phases. For oxides, during calcination, the thermal diffusion of ions is enhanced, making the formation of vacancies energetically favorable in certain configurations [55].

This phenomenon may be more pronounced when there is a disproportion between cations and anions in the structure [56,57]. New vacancies can form in close proximity to one another due to the trapping of electrons in already existing vacancies, intensifying the black coloration of the scaffolds. Studies [58,59] indicate that the presence of polyvinylpyrrolidone (PVP) can influence the formation of oxygen vacancies in TiO_2_ during thermal treatment in oxygen-rich atmospheres. Additionally, there is evidence that carbon can promote the formation and stabilization of oxygen vacancies in materials containing boron and silicon [60,61].

For the doped glasses, the black coloration is believed to be more pronounced due to an increase in vacancies. This effect may result from the partial substitution of metallic cations in the vitreous network by Zn^2^⁺ and Mg^2^⁺ ions, which can create an imbalance in the cation-anion ratio [62,63,64]. Furthermore, the addition of hydrated precursors contributes to a greater release of gases, such as NO_2_ and H_2_O, during the decomposition of nitrates. This can intensify the formation of vacancies and, consequently, the black coloration of the doped glasses. However, the powder resulting from the sol-gel synthesis of the pure 45S5 glass, without the presence of PVP, exhibited a common white coloration.

Oxygen vacancies play a crucial role in photothermal therapy, as they are responsible for generating non-radiative heat from the absorbed energy. When the material absorbs near-infrared (NIR) light, photons with energy equal to or greater than the bandgap collide at the surface, promoting the transfer of electrons from the valence band to the conduction band and creating vacancies. These vacancies prevent the natural recombination of photoelectrons, generating the heat necessary to destroy cancer cells [65]. The increase in vacancy concentration in scaffolds can enhance the efficiency of photothermal therapy, as studies [66,67] show that a higher concentration of vacancies leads to greater absorption of near-infrared (NIR) light. This, in turn, improves the conversion of light energy into heat, making the material more effective for photothermal therapy applications.

**Figure 2:**
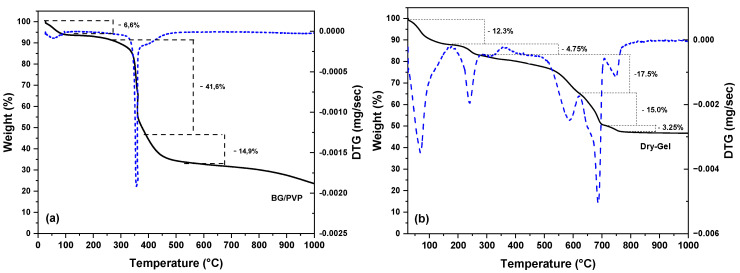
Thermogravimetric analysis (TGA) (black line) and derivative thermogravimetry (DTG) (blue line) of (**a**) the pure fiber (BG-PVP) and (**b**) the dry gel of the powder (Dry-Gel).

The thermogravimetric (TGA) and derivative thermogravimetric (DTG) curves (Figure 2) showed three events of mass loss for the fibrous sample, while five events were observed for powders obtained by the sol-gel route. In fibrous samples, the first event occurred in the temperature range from room temperature to approximately 130 °C, indicating the elimination of adsorbed water and alkoxide groups, beginning at room temperature [68,69] with an initial mass loss of 6.6%. The second event took place between 260 °C and 380 °C, resulting in a loss of 41.6%, followed by a third event occurring in the temperature range of 380 °C to 550 °C, with a loss of 14.9%. These events can be attributed to the decomposition of PVP, which typically occurs in two stages: one in the range of 250 °C to approximately 440 °C, related to the elimination of the vinylpyrrolidone group, and another between approximately 440 °C and 550 °C, reflecting the complete decomposition of the polymer chains [70,71]. Furthermore, these events may also be associated with the thermal decomposition of the nitrates used as precursors for the oxides in the synthesis process, as observed in previous studies [72,73].

Events occurring around 460–530 °C may be related to the decomposition of the nitrate ion (NO_3_⁻) [74,75]. Studies indicate that the thermal decomposition of metal nitrates can occur at lower temperatures in the presence of hydrogen [76,77]. Thus, the decomposition of sodium nitrate may have been facilitated by the weakening of the bonds between the Na⁺ ion and the nitrate anion (NO_3_⁻), caused by the OH bonds from the polymer chains of PVP. The hydroxyl (OH) groups of PVP can release hydrogen gas (H_2_) during the decomposition process in the presence of carbon [78]. These interactions create a reducing environment for nitrogen, resulting in the release of gases and water (2 NaNO_3_ + H_2_ → Na_2_O + N_2_ + H_2_O), which consequently reduces the decomposition temperature. Therefore, the curve suggests that mass loss of events occurs before reaching 600 °C. The doped fibrous scaffolds exhibited similar thermal behavior.

For the dry gel sample, five mass loss events were identified in the TGA and DTG curves (Figure 2b). The first event, which occurred from room temperature up to approximately 180 °C, corresponds to the removal of water absorbed on the surface of the gel [79], representing a 12.3% mass loss. The second event, extending up to around 250 °C, is associated with the elimination of water resulting from the condensation of precursors and catalysts [80,81], with a mass loss of 4.75%. The last three events, occurring consecutively between 450 °C and 780 °C, are related to the elimination of nitrates, with mass losses of 17.5%, 15%, and 3.25%, respectively. The final event characterized the decomposition of unreacted sodium nitrate (2NaNO_3_ → Na_2_O + 2NO + 1.5O_2_), which typically occurs at high temperatures, in the range of 700–900 °C [73,82]. Therefore, to stabilize the glass powder, a temperature above 780 °C is required to ensure the complete removal of precursor materials.

**Figure 3:**
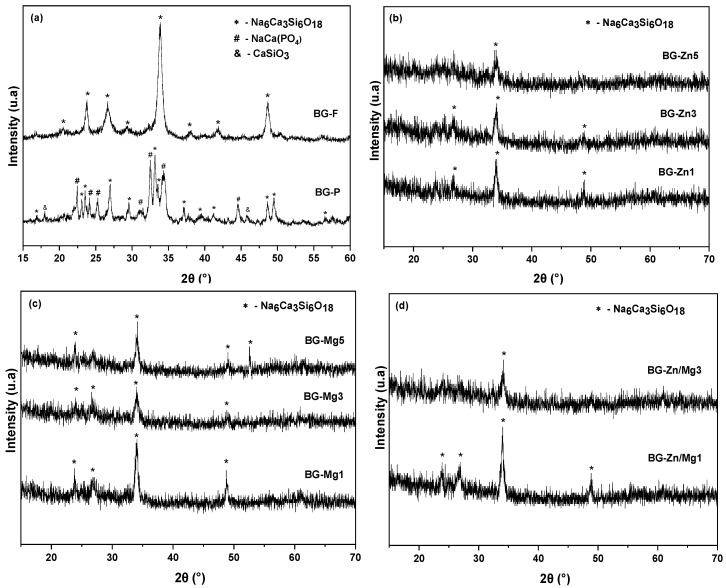
X-ray diffraction (XRD) patterns of the bioactive glass samples after calcination/stabilization: (**a**) BG-F and BG-P, (**b**) BG-Zn1, BG-Zn3, and BG-Zn5, (**c**) BG-Mg1, BG-Mg3, and BG-Mg5, (**d**) BG-Zn/Mg1 and BG-Zn/Mg3.

The X-ray diffraction (XRD) patterns of the pure nanofibrous scaffolds and the 45S5 glass powder after calcination/stabilization. (Figure 3a), presented peaks indicative of the Na_2_CaSi_2_O_6_ phase (combeite—JCPDS no. 01-077-2189), such as observed in other studies on 45S5 [80,83,84,85,86]. This process may have been facilitated by surface crystallization, promoted by the ionic nature of the alkalis present in bioactive glasses, which disrupts the glassy network [83]. Combeite, as the only crystalline phase in vitro ceramics, can provide enhanced mechanical properties associated with bioactivity [87,88]. However, this crystallization may lead to irregular degradation of the structure and uncontrolled release of ions [89].

For the powder sample BG-P (Figure 3a), in addition to the combeite phase, peaks corresponding to the NaCa(PO_4_) phase (JCPDS no. 01-076-1456) and CaSiO_3_ (wollastonite—JCPDS no. 042-0547) were identified. These phases are quite common in vitro ceramics sintered at high temperatures [90,91,92,93,94,95]. The separation of immiscible phases can be explained by the presence of different ionic species, such as Si^4^⁺ and P^5^⁺, which lead to the formation of specific rich phases [96]. Although this increase in crystallinity benefits mechanical properties, it may reduce the dissolution of ions from the glassy network, negatively affecting biocompatibility [97].

The addition of Mg and Zn ions to the fibrous scaffolds resulted in a decrease in the crystallization of the glasses (Figure 3b–d), both in the doping and co-doping across all studied compositions. One study showed studies [98] that the presence of Mg and Zn doping ions reduced the crystallization of the nano bioceramic. Furthermore, research [99,100,101] demonstrated that substituting Ca with small amounts of Mg^2^⁺ and Zn^2^⁺ improves the synthesis of 45S5 glass. According to Wetzel et al. (2020) [38], this occurs due to the higher field strength of Mg^2^⁺ and Zn^2^⁺ compared to Ca^2^⁺, which attracts oxygen ions more strongly, stabilizing the glassy network and making the necessary rearrangement for crystallization more difficult, resulting in a broader processing window.

**Figure 4:**
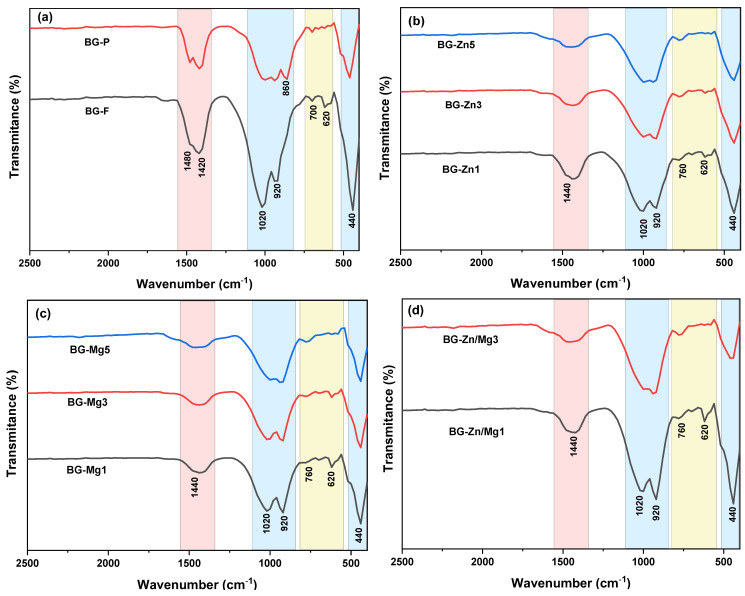
FTIR spectra of the glasses: (**a**) BG-F and BG-P, (**b**) BG-Zn1, BG-Zn3, and BG-Zn5, (**c**) BG-Mg1, BG-Mg3, and BG-Mg5, (**d**) BG-Zn/Mg1 and BG-Zn/Mg3. The blue areas correspond to the silicate group, the pink to ionic carbonates, and the yellow to the phosphate group.

The FTIR spectra of the doped samples were quite similar to those of the pure samples (Figure 4). More pronounced bands are observed in the fibrous samples at approximately 1020 cm^−1^ and 920 cm^−1^, corresponding to the symmetric and asymmetric stretching vibrations of Si–O–Si, along with a band around 440 cm^−1^, indicating the asymmetric stretching of the same group [91]. The peak at 860 cm^−1^ present in the BG-P sample (see Figure 4a) refers to the Si–O stretching, which is attributed to the silicon bond in the phosphorus-rich phase according to the literature [102]. Additionally, the peak at 700 cm^−1^ corresponds to the bending vibrations of Si–O groups present on the surface of the glassy silica [103,104]. Small bands around 620 cm^−1^ are attributed to the bending vibrations of the P–O group, related to the phosphoryl groups in the structures [105].

Furthermore, bands corresponding to purely ionic carbonates (CO_3_^2^⁻) were identified around 1480 cm^−1^ and 1420 cm^−1^, attributed to C–O vibrations, resulting from the high reactivity of the surfaces of the bioactive glasses during stabilization. Studies show that the interaction between the O–H groups (band at 3200 cm^−1^), present due to excess water in the structure, and atmospheric CO_2_ on the surface of the samples favors the carbonation reaction: CO_2_ + H_2_O → CO_3_^2^⁻ + 2H⁺ [106,107], impacting the bioactive properties of the samples.

**Figure 5:**
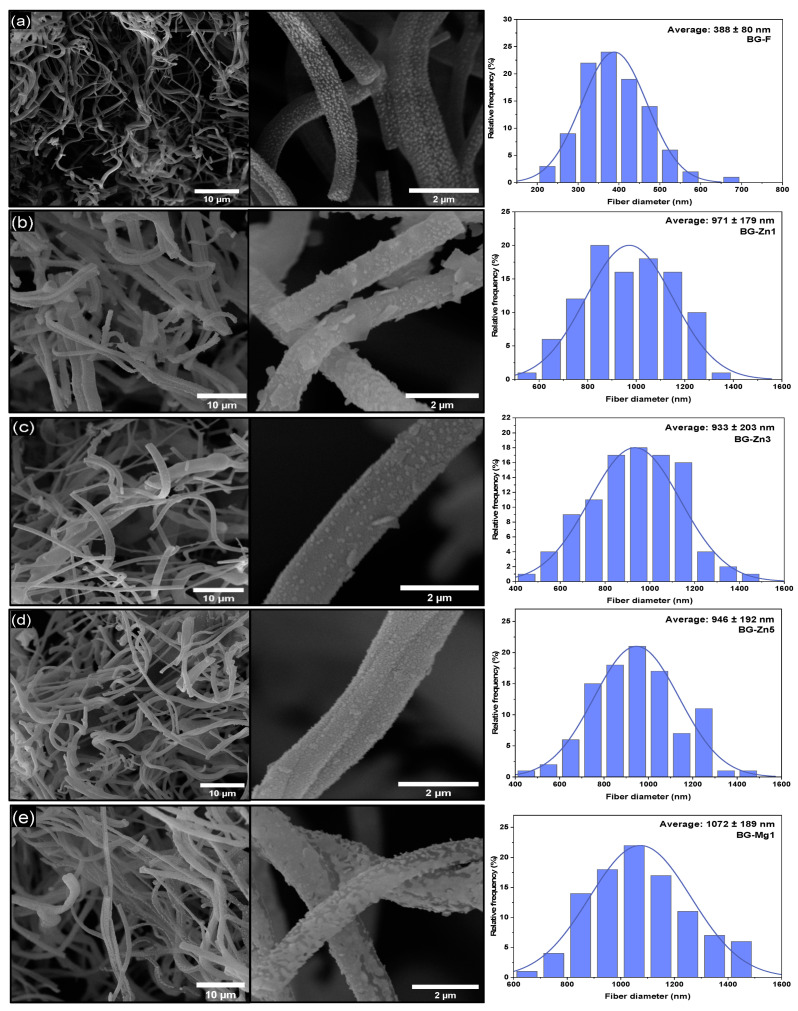

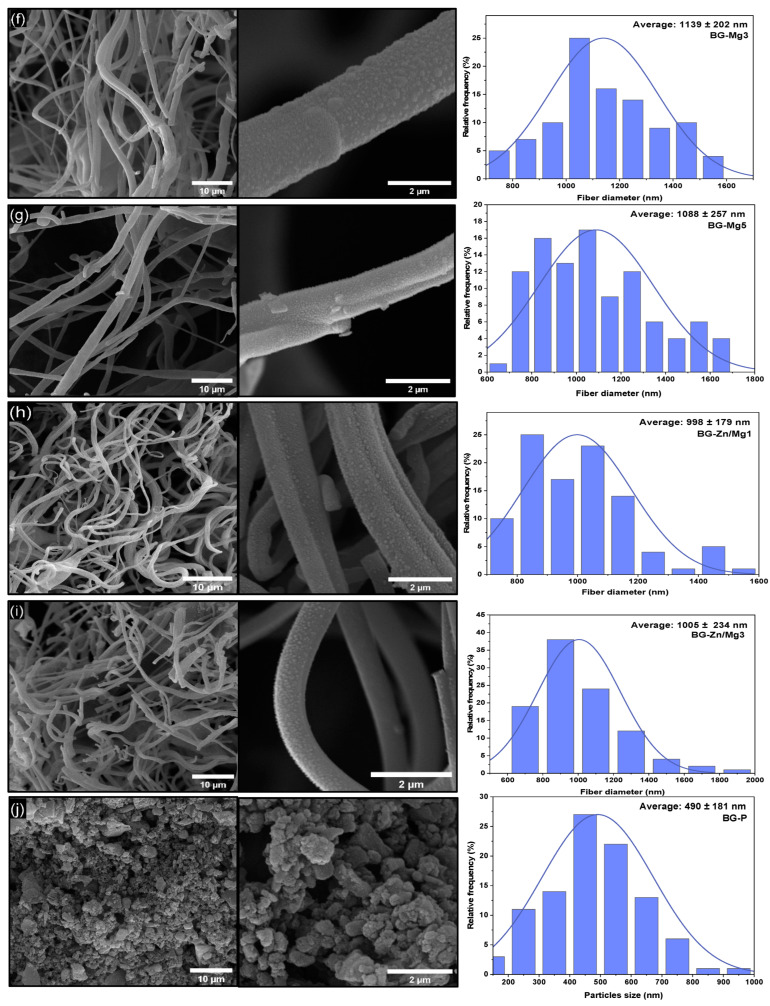
SEM images and average size distribution of BG-F (**a**), BG-Zn1 (**b**), BG-Zn3 (**c**), BG-Zn5 (**d**), BG-Mg1 (**e**), BG-Mg3 (**f**), BG-Mg5 (**g**), BG-Zn/Mg1 (**h**), BG-Zn/Mg3 (**i**), and BG-P particles (**j**).

Figure 5 illustrates the micrographs and fiber diameter distribution graphs of the nanofibrous scaffold fibers. SEM analyses revealed the formation of continuous fibers arranged randomly for the pure fibrous sample (BG-F) as well as for the doped and co-doped samples. Table 2 presents the average diameter values of all fibrous samples along with the results of the Tukey test. According to this test, there were significant differences between the average diameters of the pure fiber, which measured 388 ± 80 nm, and all doped samples with Zn and Mg, regardless of concentration, which exhibited micrometric fibers (*p* < 0.05). However, no significant differences were observed among the samples in the Zn doping group (BG-Zn1, BG-Zn3, and BG-Zn5), in the Mg group (BG-Mg1, BG-Mg3, and BG-Mg5), and in the co-doped samples (BG-Zn/Mg1 and BG-Zn/Mg3) (*p* > 0.05).

This difference may be attributed to the higher viscosity of the solutions of the doped systems, resulting from the electrostatic interactions between the ions and the polymer’s molecules [108,109]. The high charge of the added dopant ions, combined with their small atomic radii, increases the electrostatic interactions, making it difficult for the polymer chains to move. Thus, it is believed that during the spinning process, there was greater resistance to the stretching of the solutions, resulting in larger fiber diameters [110]. It has been reported in the literature [111] that the substitution of calcium with dopant and co-dopant ions in bioactive glasses led to a significant increase in fiber diameters, even at low concentrations, when using the electrospinning method.

Figure 5j shows the morphology and particle size distribution graphs of the synthesized powder. Particles of varied sizes, irregular shapes, and non-uniformity can be observed, as reported in previous studies [69,79,112,113,114]. The average particle size was 490 ± 181 nm, although larger particles, ranging from 1 to 5 µm, were present due to agglomeration. Additionally, larger plate-like structures were observed, which may be related to the formation of crystals in the combeite phase, as suggested in literature [115].

The porosity of the scaffolds, determined using the Archimedes method (Equation (3)), ranged from 90 to 93%. The porosity of doped scaffolds did not show a statistically significant difference from the porosity of the pure sample. In this way, pure and doped scaffolds exhibited characteristics similar to bioactive hydrogel structures. This is very interesting for biological applications as they promote nutrient and oxygen exchange, waste removal, and the growth of bone and vascular tissue through the scaffold, playing a crucial role in bone regeneration [116,117,118,119].

**Figure 6:**
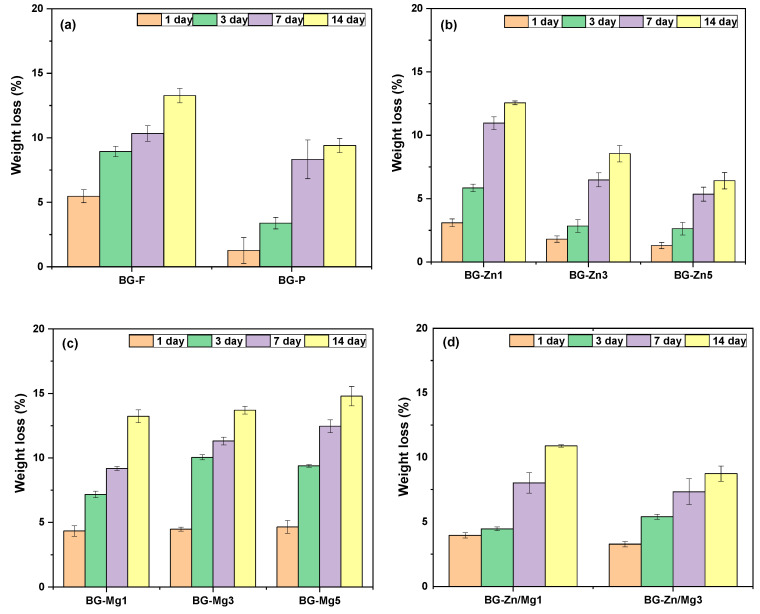
Degradation profile of the glasses: (**a**) BG-F and BG-P, (**b**) BG-Zn1, BG-Zn3, and BG-Zn5, (**c**) BG-Mg1, BG-Mg3, and BG-Mg5, (**d**) BG-Zn/Mg1 and BG-Zn/Mg3.

The degradation profile shown in Figure 6 was evaluated by immersing the bioactive glasses (BGs) in PBS solution for 14 days. All samples exhibited loss of mass over this period, confirming the biodegradable behavior of bioactive glasses. The pure fibrous scaffold and powder showed a gradual weight loss pattern, with the fibrous sample displaying a higher loss of 13% and the powder showing a 9% loss after 14 days. This result may be related to the higher surface area of the fibers, which enhances interaction with the medium and accelerates the degradation process [120]. Additionally, studies indicate that the crystallinity of 45S5 glass improves structural stability and tends to hinder interaction with the medium [73,92].

The zinc concentrations in the doped scaffolds led to a decrease in mass loss (Figure 6b). After 14 days, the mass losses were 12%, 8%, and 6% for the zinc concentrations of 1%, 3%, and 5%, respectively. This suggests that increasing the zinc concentration in the scaffolds reduces the degradation rate. Studies [121,122,123] reported that zinc can inhibit the breakdown of the silicate network, reducing ion leaching and slowing degradation. This effect may be related to the strong binding of zinc to the silicate network, inhibiting the rapid exchange of Na^+^ ions [124]. In some cases, the presence of zinc can increase the cross-linking of the glass, forming Si−O−Zn units [125].

The magnesium-doped scaffolds exhibited similar mass loss as the Mg concentration increased, resulting in total losses of 13%, 13%, and 14% for the magnesium concentrations of 1%, 3%, and 5%, respectively (Figure 6c). This suggests that Mg ions do not negatively interfere with the dissolution and degradation of the scaffolds. Studies [126,127] indicate that the partial substitution of CaO with MgO can intensify weight loss, promoting the formation of an apatite layer on the glass surface. The co-doped scaffolds (Figure 6d) showed mass losses of 10% and 8% for the Zn/Mg1 and Zn/Mg3 samples, respectively, after 14 days. This suggests that the combined presence of zinc and magnesium ions at higher concentrations (3%) tended to reduce scaffold degradation. The lower degradation rate may be related to the higher presence of zinc.

**Figure 7:**
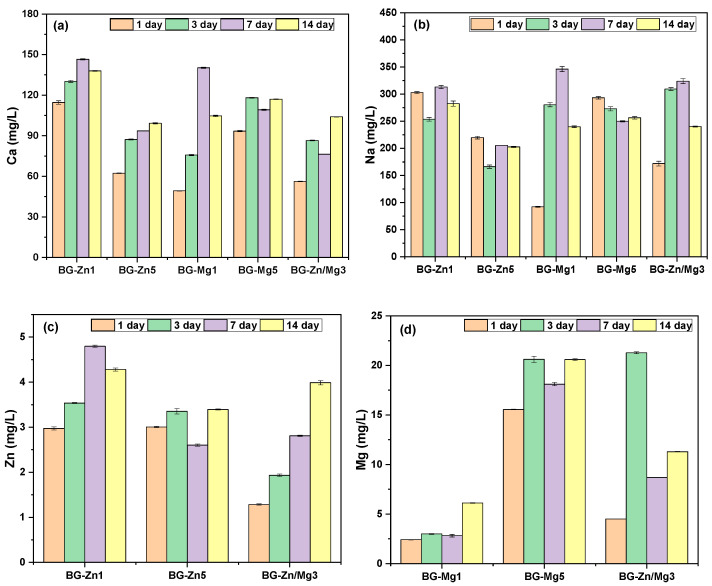
Ionic release in TRIS from the samples BG-Zn1, BG-Zn5, BG-Mg1, BG-Mg5, and BG-Zn/Mg3: (**a**) Ca, (**b**) Na, (**c**) Zn, and (**d**) Mg.

The release of Ca, Na, Mg, and Zn ions from the BG-Zn1, BG-Zn5, BG-Mg1, BG-Mg5, and BG-Zn/Mg3 samples is shown in Figure 7. Ca release was highest in the 1% doped samples on day 7, with 146 mg/L for BG-Zn1 and 140 mg/L for BG-Mg1, while higher dopant concentrations showed lower release (Figure 7a). Previous studies [128,129,130] suggest that Ca^2+^ release at 2–4 mM (1 mM equals 180 mg/L) concentrations promotes osteoblast proliferation and survival, with cytotoxicity occurring only at higher levels.

Na release was similar across all compositions, being lowest in the higher Zn concentrations, with a maximum value of 219 mg/L (Figure 7b). These values align with literature for 45S5 glass [131]. Na release affects the pH, which may create unfavorable conditions for bacterial growth or reduce the glass’s cytocompatibility, though it can also influence alkaline phosphatase (ALP) activity and impact osteogenic cell differentiation [131].

The release of Zn was similar in BG-Zn1 and BG-Zn5, ranging from 2.9 to 4.28 mg/L and 3 to 3.39 mg/L, respectively, over time. The highest concentration was reached on day 7 for BG-Zn1 and on day 14 for BG-Zn5. In the co-doped sample (BG-Zn/Mg3), Zn release had a wider range, from 1.29 to 3.98 mg/L (Figure 7c), with a peak on the 14th day. These levels suggest that even at higher concentrations, Zn release may not cause cytotoxic effects, as indicated in the literature [132]. Studies show that Zn release between 2.45 and 6.5 ppm (mg/L) promotes bone formation in vitro and in vivo [133,134], and concentrations between 3 and 7 ppm (mg/L) are effective for antibacterial action [135]. However, higher concentrations may induce cytotoxicity [136].

In the literature, the minimum inhibitory concentration (MIC) values for Zn^2+^ ions in vitro are relatively high. For Gram-negative bacteria, such as *Escherichia coli*, *Klebsiella pneumoniae*, *Acinetobacter baumannii*, *Pseudomonas aeruginosa*, and *Enterobacter cloacae*, MIC values range from 1 to 4 mM (24.3 to 97.2 mg/L) [137]. In the case of zinc oxide nanoparticles (ZnONPs), the reported MIC against *S. aureus* and *S. Typhimurium* was 0.05 mg/mL (50 mg/L) [138]. Based on the cumulative zinc release values observed throughout the experiment, the concentrations obtained—15.57 mg/L (BG-Zn1), 12.34 mg/L (BG-Zn5), and 10 mg/L (BG-Zn/Mg3)—may be insufficient for effective antibacterial action, considering the higher MIC values reported.

The BG-Mg5 sample exhibited the highest Mg release, ranging from 15.55 to 20.61 mg/L, while BG-Mg1 showed a lower and more stable range, varying from 2.42 to 6.13 mg/L, with both peaks occurring on the 14th day. In the co-doped sample (BG-Zn/Mg3), Mg release varied from 4.51 to 11.29 mg/L, peaking at 21.27 mg/L on day 3 (Figure 7d). According to Wang et al. (2015) [139], Mg release in alkaline conditions can be beneficial or toxic depending on the cell type: in L929 cells and osteoblasts, levels up to 35 mM did not cause cytotoxicity, while the safe limit for BMSCs and MC3T3-E1 cells is up to 15 mM.

Magnesium (Mg^2+^) is not commonly used for antibacterial activity since, as reported in the literature, high ion concentrations (MIC) are required for this effect to be observed. The following studies indicate that Mg^2+^ exhibits little to no antimicrobial activity at typical concentrations. According to Nguyen, et al. [140], transiently increased Mg^2+^ ion concentrations ranging from 1 to 50 mM (24.3 to 1215 mg/L) showed no inhibitory or bactericidal effect against Staphylococcus epidermidis. Similarly, studies conducted by Xie and Yang [141] established that the minimum dose required to eliminate a significant percentage of inoculated Staphylococcus aureus cells is 20 mM (486 mg/L). Additionally, Mg^2+^ had minimal impact on the viability of Escherichia coli and Bacillus subtilis.

Thus, the cumulative release values of the samples—14.38 mg/L (BG-Mg1%), 74.86 mg/L (BG-Mg5%), and 45.75 mg/L (BG-Zn/Mg3)—are insufficient to reach the MIC values necessary to confer significant antibacterial activity.

Studies [142] observed that the substitution of zinc for calcium in 45S5 bioactive glasses can significantly reduce the release of ions in Tris-HCl solution, resulting in lower ionic exchanges. Conversely, the increase in magnesium concentration allowed for a more balanced release. One possible explanation for this behavior is that the field strength of the Zn^2+^ ion is greater than that of Ca^2+^, since zinc has an atomic radius of 0.60 Å and is generally in tetrahedral coordination (4), while calcium, with a larger ionic radius of 1.0 Å, tends to have octahedral coordination (6) [143]. Thus, the smaller ionic radius, combined with the stronger bond strength of zinc in the glass network, may reduce its ionic mobility, directly impacting its release.

Even though the atomic radii of Zn^2+^ and Mg^2+^ ions are quite similar, magnesium likely acts in the network as a modifier, as expected [144]. Zinc, on the other hand, appears to behave as an intermediate cation, affecting not only its own release but also the release of other modifier ions, such as calcium and sodium, thereby impacting the overall release dynamics of the scaffolds [142,145,146,147,148].

**Figure 8:**
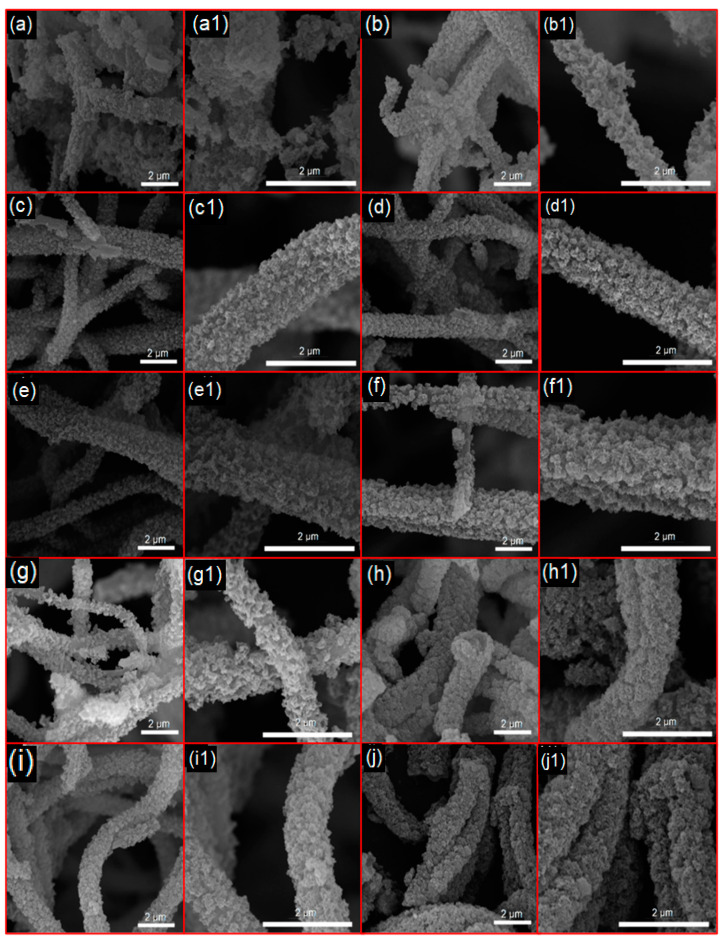
SEM images after immersion in SBF of BG-F at 24 h (**a**,**a1**) and 72 h (**b**,**b1**), BG-Mg1 at 24 h (**c**,**c1**) and 72 h (**d**,**d1**), BG-Mg3 at 24 h (**e**,**e1**) and 72 h (**f**,**f1**), BG-Mg5 at 24 h (**g**,**g1**) and 72 h (**h**,**h1**), and BG-Zn/Mg1 at 24 h (**i**,**i1**) and 72 h (**j**,**j1**).

**Figure 9:**
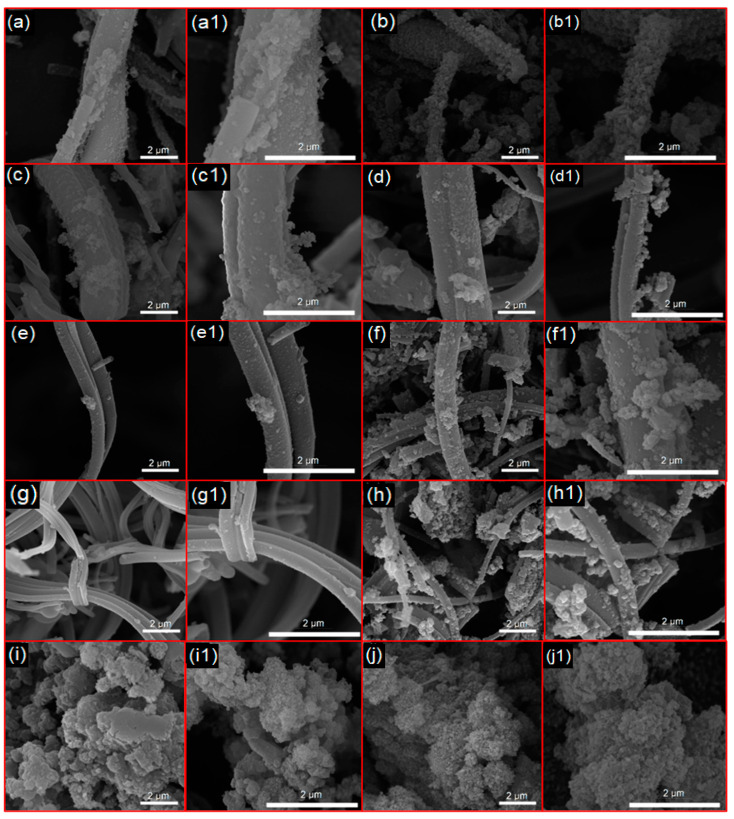
SEM images after immersion in SBF of BG-Zn1 at 24 h (**a**,**a1**) and 72 h (**b**,**b1**), BG-Zn3 at 72 h (**c**,**c1**) and 168 h (**d**,**d1**), BG-Zn5 at 72 h (**e**,**e1**) and 168 h (**f**,**f1**), BG-Zn/Mg3 at 72 h (**g**,**g1**) and 168 h (**h**,**h1**), and BG-P at 24 h (**i**,**i1**) and 72 h (**j**,**j1**).

**Figure 10:**
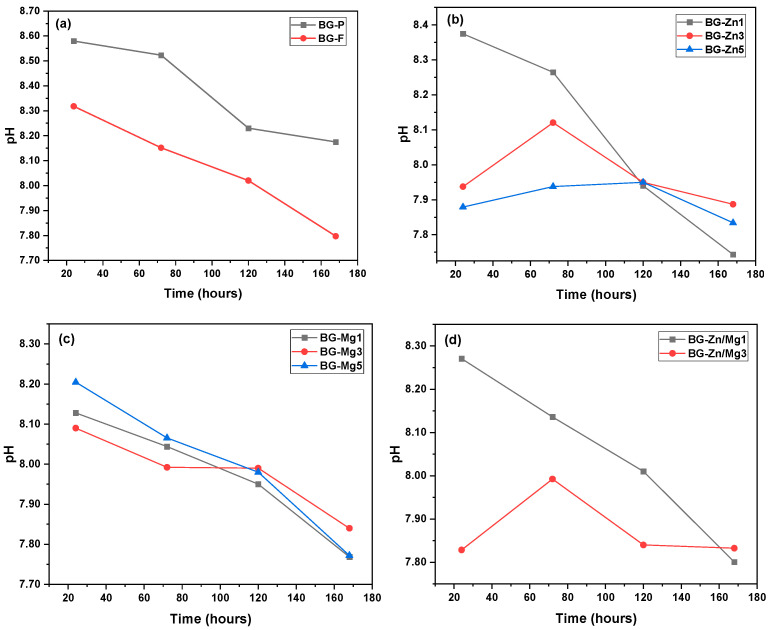
pH variations during different immersion times in SBF for up to 168 h of the glasses: (**a**) BG-F and BG-P, (**b**) BG-Zn1, BG-Zn3, and BG-Zn5, (**c**) BG-Mg1, BG-Mg3, and BG-Mg5, and (**d**) BG-Zn/Mg1 and BG-Zn/Mg3.

**Figure 11:**
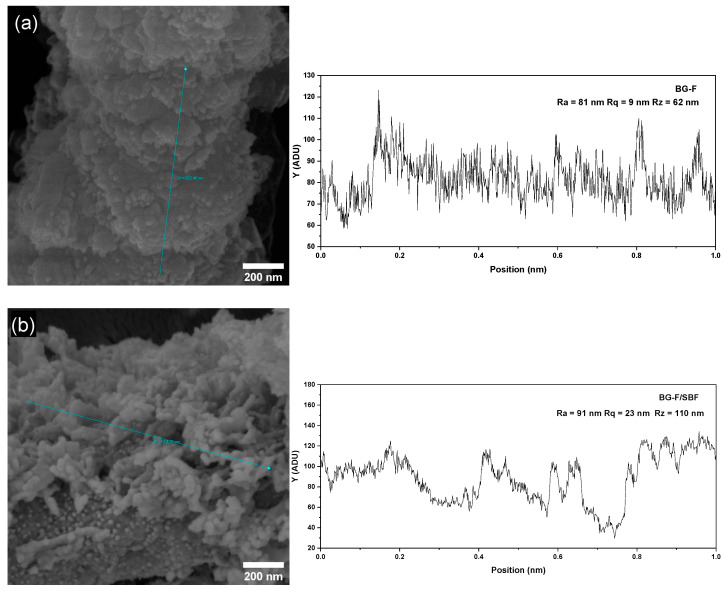
FEG images and microroughness profile along a 1 µm line (blue line) on the surface of the BG-F sample before (**a**) and after 168 h of immersion in SBF (**b**).

**Figure 12:**
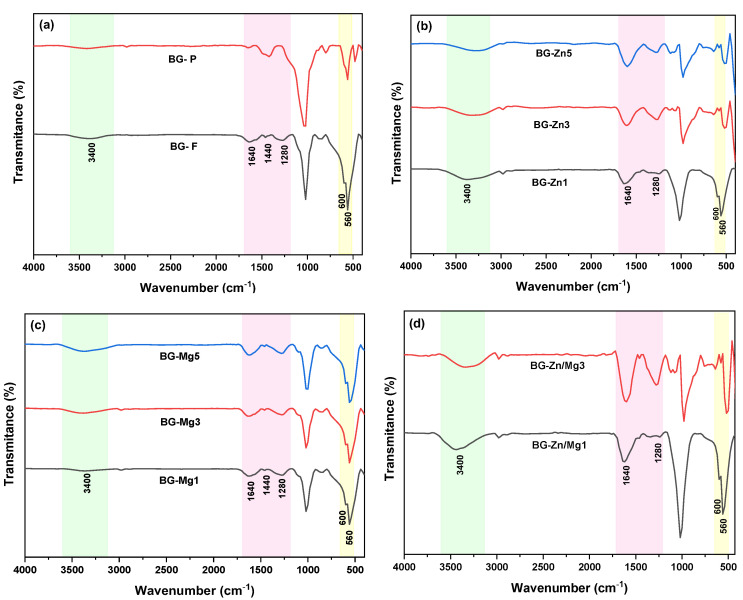
FTIR spectra after 168 h of immersion in SBF of the glasses: (**a**) BG-F and BG-P, (**b**) BG-Zn1, BG-Zn3, and BG-Zn5, (**c**) BG-Mg1, BG-Mg3, and BG-Mg5, and (**d**) BG-Zn/Mg1 and BG-Zn/Mg3. The green area highlights the hydroxyl group, the pink area the carbonate group, and the yellow area the phosphate group.

The bioactivity of the bioactive glasses (BGs) was assessed after immersion in simulated body fluid (SBF), with the formation of hydroxyapatite confirmed through MEV, FTIR, and XRD analyses (Figure 8, Figure 9, Figure 10, Figure 11 and Figure 12). The micrographs of the fibrous scaffolds and the powder after immersion can be seen in Figure 8 and Figure 9. After 24 h, the formation of apatite crystals in spherical clusters was observed, distributed across most of the surfaces of the BG-F, BG-Mg1, BG-Mg3, BG-Mg5, and BG-Zn/Mg1 samples (Figure 8), with initial formation noted in BG-Zn1 and BG-P (Figure 9). On the third day, a homogeneous precipitation resembling cauliflower completely covered the surfaces of these scaffolds. However, in the zinc-doped samples BG-Zn3, BG-Zn5, and BG-Zn/Mg3 (Figure 9), apatite formation began only after 72 h, with a gradual increase observed at 168 h.

These results confirm studies by Du et al. (2006) [149], which indicate that higher concentrations of zinc may delay the nucleation of hydroxyapatite during the initial immersion but do not affect its long-term formation. On the other hand, the presence of Mg^2+^ may promote the development of a thicker layer of hydroxyapatite (HA), due to greater leaching of ions from the surface as the MgO content in the glass increases, as noted by Oliveira et al. (2002) [150].

When analyzing the pH values of the BGs (Figure 10), it is noticeable that the samples showed significant increases within the first 24 h of immersion, except for those with higher zinc concentrations, which reached their highest pH peaks after 72 h. Over the immersion period, the pH gradually decreased, stabilizing below 7.9.

The fibrous scaffolds tended to generate a moderately alkaline pH, which may favor the antibacterial effect. Studies have shown that the pH increase caused by 45S5 glass can promote antibacterial activity by altering the bacterial membrane and disrupting intracellular ions, leading to bacterial cell death [151,152,153].

Although alkaline phosphatase activity, associated with osteogenesis and cell proliferation, was found to be ideal in the pH range between 7.6 and 7.8 [154,155], other studies have suggested that a slight pH increase from 7.4 to 8.0 can significantly enhance mineralization and improve mesenchymal stem cells (MSC) differentiation into osteoblasts. However, increasing the pH to 8.5 did not further enhance differentiation [156], and osteoblast-like cells (MC3T3-E1) showed maximum proliferation in the pH range of 8.0 to 8.4, although prolonged exposure should be monitored [157].

Furthermore, studies by Karkozar et al. (2022) [33] showed that in alkaline environments (pH ~ 8.2), magnesium-doped glasses promoted calcium deposition in osteoblastic cells, such as MG-63 cell lines, after 14 days. The lower pH values in the zinc samples may be more favorable for osteoblast proliferation, while the 45S5 powder appears less favorable due to its higher pH values. These findings highlight the importance of further research on the alkaline range for osteoblast proliferation.

This variation in pH results from the ion exchange between the ions in the samples and the SBF solution, which initially had a pH of 7.40. The Ca^2+^ and Na^+^ ions are released from the surface of the bioactive glass and exchanged for H_3_O⁺ ions from the solution [158]. This process increases the concentration of hydroxyl ions (OH^−^), raising the pH of the medium [159]. The increase in pH promotes the breaking of Si–O–Si bonds in the glass structure, forming silanol groups (Si–OH) on the surfaces of the samples. These silanol groups are essential for the formation of the hydroxyapatite (HA) layer. Therefore, the greater the leaching of ions, the more effective the formation of the HA layer, which is crucial for the bioactivity of bioactive glasses [160].

The decrease in pH over the immersion time reflects the nucleation and growth of hydroxyapatite (Ca_10_(PO_4_)_6_(OH)_2_), involving the combination of Ca^2+^ and OH^−^ ions with PO_4_^3−^ ions. As these ions are consumed, the concentration of free ions in the solution decreases, resulting in a drop or stabilization of the pH [161,162]. This behavior indicates that the ions are being progressively removed from the solution and incorporated into the structure of hydroxyapatite, forming an increasingly dense and continuous layer of HA on the surface of the bioactive glass [163,164].

The surface roughness of the scaffolds was analyzed using FEG images of the BG-F samples before (a) and after immersion in SBF (b), along with their respective intensity profile graphs (Figure 11). The images clearly show the microroughness of the scaffolds, highlighting the deposition of hydroxyapatite on the fiber surfaces. Based on the graphs, the roughness parameters obtained from the profile line analysis were BG-F (Ra = 81 nm; Rq = 9 nm; Rz = 62 nm) and BG-F/SBF (Ra = 91 nm; Rq = 23 nm; Rz = 110 nm), indicating that the scaffolds maintained their microscale roughness even after immersion in SBF. These values align with those reported in the literature, where surface roughness at the micro and submicroscale promotes enhanced osteoblast differentiation and the production of local factors in vitro [165]. Additionally, studies suggest that controlled microroughness can stimulate the osteogenic differentiation of bone marrow mesenchymal stem cells on surfaces with Ra values of up to 2–3 μm [166].

Figure 12 presents the FTIR spectra of the fibrous scaffolds and the powder after 168 h of immersion in SBF. When comparing these spectra to those taken before immersion, a notable absence or significant reduction of peaks between 560 and 415 cm^−1^ is observed, which are attributed to Si–O–Si vibrations, indicating that the silica-rich layer has polymerized [167]. At the same time, there is an intensification of the peaks around 560 and 600 cm^−1^, corresponding to the bending and stretching vibrations of P–O from the phosphate group (PO_4_^3−^), highlighting the formation of phosphates related to hydroxyapatite (HA) [168].

A broad band around 3400 cm^−1^ is also observed, associated with the presence of hydroxyl groups, along with an increase in the intensity of the bands around 1440 cm^−1^, and the emergence of new bands at 1640 and 1280 cm^−1^, related to the stretching of C–O from the carbonate group (CO_3_^2−^), suggesting the formation of hydroxycarbonate apatite (HCA) [169]. This form of apatite, structurally similar to the apatite found in bones, facilitates integration with living tissues, making it ideal for applications in bone prosthetics [170,171]. Samples with higher zinc content exhibited discrete peaks, suggesting a lower amount of HA formed.

**Figure 13:**
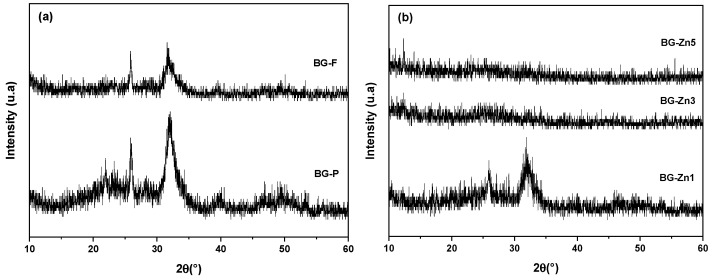

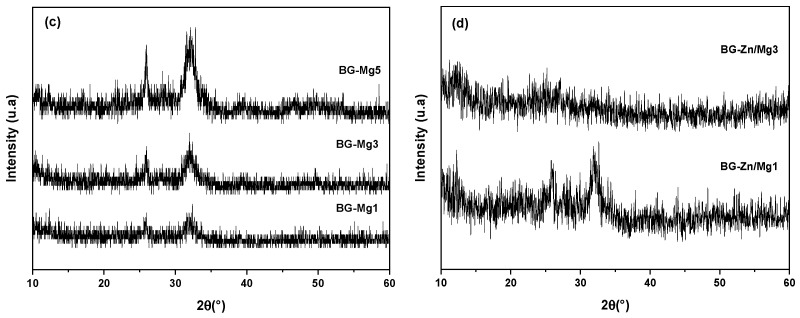
X-ray diffraction patterns after 168 h of immersion in SBF of the glasses (**a**) BG-F and BG-P, (**b**) BG-Zn1, BG-Zn3, and BG-Zn5, (**c**) BG-Mg1, BG-Mg3, and BG-Mg5, (**d**) BG-Zn/Mg1 and BG-Zn/Mg3.

Supporting the previous results, Figure 13 shows the X-ray diffraction patterns of the scaffolds after 168 h of immersion in SBF. More pronounced peaks are observed at 2θ = 31.7° and 25.8°, corresponding to the (211) and (002) planes, characteristic of hydroxyapatite formation in the hexagonal system (JCPDS No. 09-0432) [172,173] for all samples, except for those with higher concentrations of zinc. It is believed that for the samples doped with zinc at concentrations of 3% and 5% (Figure 13b,c), as well as for the co-doping with 3% (Figure 13d), the identification of HA was not possible due to the low amount formed.

Previous studies have reported that incorporating Zn into 45S5 glass increased the chemical stability of the silicate structure as its concentration rose, leading to a reduction in HAp layer formation [174]. Similarly, research conducted by Miola, et al. [175] indicated that Zn delayed HAp nucleation; however, after a few days of immersion in SBF, the formation of a silica gel was observed, followed by its enrichment with Ca and P. The delay in HAp formation has also been observed in the presence of magnesium, as the leaching of this element into the SBF solution reduces the formation rate of a more stable apatite phase. Additionally, studies have indicated that increasing the magnesium concentration in the glass tends to slow down apatite layer formation on the material’s surface [176,177].

In this study, the effects of Zn were consistent with literature data, confirming the delay in apatite formation. However, the Mg-doped scaffolds exhibited a distinct behavior, and Mg ions did not hinder hydroxyapatite formation. The results suggest that, depending on the amount, magnesium may preserve the bioactive properties of 45S5 glass, thereby maintaining its high bioactivity.

**Figure 14:**
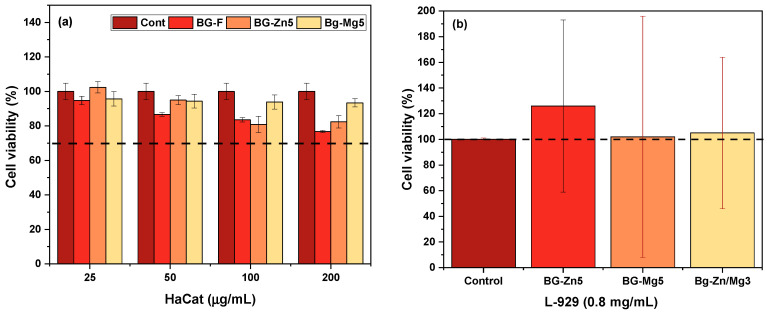
Evaluation of cell viability (**a**) of HaCaT cells after 72 h of exposure to different concentrations (25–200 µg/mL) of BG-F, BG-Zn5, and BG-Mg5 samples, and (**b**) of L929 cells after 24 h of exposure to BG-Zn5, BG-Mg5, and BG-Zn/Mg3 samples. The dashed line indicates 70% viability for HaCaT (**a**) and 100% for L929 (**b**).

The cytotoxicity of the pure scaffold (BG-F) and those doped with the highest concentration of each ion (BG-Zn5, BG-Mg5) was evaluated using the MTT assay in HaCat cells (Figure 14a). After 72 h of exposure, and at a concentration of 50 µg/mL, only BG-F reduced cell viability compared to the control group (100.0% ± 4.7), starting (86.57% ± 1.0) (*p* < 0.01). This effect may be explained by the rapid release of alkaline ions from the nanofibers into the medium, which elevates the pH [178]. In contrast, BG-Zn5 significantly reduced cell viability starting at a concentration of 100 µg/mL (80.85% ± 4.71) (*p* < 0.01), while BG-Mg5 did not alter viability, maintaining values above 90%.

Studies indicate that keratinocytes, the primary cells of the epidermis (approximately 95%), can experience cytotoxic effects due to the imbalance generated between extracellular and intracellular concentrations of Zn^2+^, affecting cell survival [179,180,181]. Studies present differing perspectives on Mg^2+^ concentrations that do not cause adverse effects. Lange et al. (1995) [182] observed that Mg^2+^ alone promotes cell migration at an optimal level of 10 mM for keratinocytes. In contrast, Grzesiak (1995) [183] reported that the ideal range for human keratinocyte migration is between 1 and 3 mM of Mg^2+^. Furthermore, Yoshino (2024) [184] suggested that concentrations as low as 0.8 mM of Mg^2+^ may lead to adverse effects, such as cellular inhibition. Thus, the release of 21 mg/L (~0.86 mM) of magnesium ions (Figure 7d) from the scaffold with the highest magnesium concentration did not compromise keratinocyte viability.

According to ISO 10993-5 [43] (cytotoxicity testing, in vitro methods), a material is considered toxic when its cell viability is less than 70%. The fibrous scaffolds exhibited non-toxic characteristics for these sample concentrations compared to the control group [178,185] in concentrations up to 200 µg/mL. Nevertheless, the dosage of the concentration is crucial for successful applications.

The cytotoxicity of scaffolds with higher dopant concentrations (BG–Zn5, BG–Mg5, and BG–Zn/Mg3) was evaluated using the MTT assay on L929 fibroblasts after 24 h of incubation (Figure 14b). All samples showed cell viability above 100% compared to the control group. The study of L929 fibroblasts is essential for evaluating the biological response of bioactive glasses, particularly regarding their ability to support cell proliferation. These fibroblasts play a critical role in wound healing and tissue repair, as they are responsible for producing extracellular matrix components and facilitating cell migration [186].

The BG–Zn5 sample exhibited the highest cell viability (126 ± 67), which could be attributed to its slower structural degradation, providing a more controlled environment for cell growth without abrupt increases in pH. These findings are consistent with previous studies demonstrating that bioactive glass nanoparticles doped with 5% zinc exhibited favorable cytocompatibility with 3T3 and L929 cells after 24 h of culture at a concentration of 80 μg/mL [187]. Furthermore, özarslan et al. (2023) [188] reported that zinc-doped 45S5 bioactive glass, when incorporated into Vaseline ointments, exhibited high levels of mitochondrial activity (>80% of the control), indicating a considerable increase in L929 fibroblast viability, particularly at higher zinc ion concentrations, such as 5 mg/mL.

Another study highlighted that partially replacing CaO with ZnO in concentrations of up to 6 wt% in electrospun PCL/ZBG bioscaffolds did not negatively affect the viability of L929 fibroblasts exposed to extracts at concentrations of 200, 20, and 2 mg/mL. On the contrary, an enhancement in cell proliferation was observed with increasing zinc content [189].

The BG-Mg5 and BG-Zn/Mg3 samples exhibited cell viability values similar to the control group, with 102 ± 94% and 105 ± 59%, respectively. Zhu et al. (2022) [190] reported that bioactive glass extracts containing varying magnesium proportions (2%, 5%, and 10%) caused no significant changes in cell density among samples after 1, 3, and 5 days. After 24 h, only a few dead cells were observed, while most L929 cells remained viable. Similarly, Daguano et al. (2013) [191] observed that the 3CaO–P_2_O_5_–SiO_2_–MgO system provided cell viability exceeding 80% in L929 fibroblasts at various extract concentrations. In contrast, Gabbai-Armelin et al. (2019) [192] reported lower viability in L929 fibroblasts and MC3T3-E1 cells exposed to glasses doped with 5% magnesium after 1 and 6 days of culture.

Thus, considering that scaffolds with higher dopant concentrations showed no cytotoxicity at an extract concentration of 0.8 mg/mL, it is reasonable to infer that the other scaffolds analyzed in this study are also non-toxic. All samples demonstrated cell viability well above the 70% threshold established by ISO-10993-5 [43,191], further supporting the biocompatibility of the developed materials.

Therefore, it was observed that the black fibrous scaffolds, made from the 45S5 glass composition, demonstrated similar or even superior results in both processing and in vitro bioactivity tests, as well as degradation, compared to the powder synthesized via the conventional sol-gel route. These scaffolds exhibit a tendency to serve as a bioactive matrix and facilitate the adhesion of osteoblastic cells. Considering this, viewing bioactivity as an indispensable characteristic for multifunctionality in tissue engineering, it is believed that these materials may hold significant potential for initial studies in phototherapy.

## 4. Conclusions

Using the air-heated solution blow spinning technique (A-HSBS) and PVP as an aid, it was possible to produce simple and efficient black 3D microfibrous scaffolds of 45S5 glass, doped and co-doped with Zn and Mg. Evidence suggests that the presence of PVP as a spinning aid favored the formation of oxygen vacancies in the glass structure, resulting in the black coloration of the fibers, with greater intensity in samples with higher dopant concentrations. Moreover, PVP contributed to the thermal stabilization of the fibers at lower temperatures. The formation of crystalline phases was less pronounced in pure fibers (BG-F) and even more reduced in doped fibers. Doping with Zn and Mg ions significantly influenced the fiber diameter, resulting in thicker and less elongated fibers while maintaining surface roughness. The presence of zinc inhibited the ionic release from the glass network, which slowed down the initial biomineralization process at higher concentrations compared to magnesium doping. Furthermore, the scaffolds showed no cytotoxic effects, and magnesium doping did not significantly affect cell viability. Therefore, the scaffolds showed promising properties for tissue engineering applications, with high bioactivity, which could enable further investigations into their interaction with specialized cells, such as osteoblasts and tumor cells, as well as their antibacterial and photothermal activity, exploring their multifunctionality.

## Figures and Tables

**Table 1:** Compositions of bioactive glasses 45S5 (mol %).

Nome	SiO_2_	P_2_O_5_	CaO	Na_2_O	ZnO	MgO
BG	46.14	2.60	26.91	24.35	-	-
BG-Zn1	46.14	2.60	25.91	24.35	1.00	-
BG-Zn3	46.14	2.60	23.91	24.35	3.00	-
BG-Zn3	46.14	2.60	21.91	24.35	5.00	-
BG-Mg1	46.14	2.60	25.91	24.35	-	1.00
BG-Mg3	46.14	2.60	23.91	24.35	-	3.00
BG-Mg5	46.14	2.60	21.91	24.35	-	5.00
BG-Zn/Mg1	46.14	2.60	24.91	24.35	1.00	1.00
BG-Zn/Mg3	46.14	2.60	20.91	24.35	3.00	3.00

**Table 2:** Average diameter of the fibers and comparative values from the Tukey test.

Name	Average Diameter ± Standard Deviation (nm)	Tukey Comparison with Pure Fiber (*p*-Value)	Comparison Between Doping (*p*-Value)
BG-F	388 ± 80	-	-
BG-Zn1	971 ± 179	*p* < 0.001	*p* = 0.910 (Zn3) *p* = 0.993 (Zn5)
BG-Zn3	933 ± 203	*p* < 0.001	*p* =0.910 (Zn1) *p*= 0.999 (Zn3)
BG-Zn5	946 ± 192	*p* < 0.001	*p* =0.910 (Zn1) *p*= 0.999 (Zn3)
BG-Mg1	1072 ± 189	*p* < 0.001	*p* = 0.281 (Mg3) *p* = 0.999 (Mg5)
BG-Mg3	1139 ± 202	*p* < 0.001	*p* = 0.281 (Mg3) *p* = 0.652 (Mg5)
BG-Mg5	1088 ± 257	*p* < 0.001	*p* = 0.999 (Mg1) *p* = 0.652 (Mg3)
BG-Zn/Mg1	998 ± 179	*p* < 0.001	*p* = 1 (Zn/Mg3)
BG-Zn/Mg3	1005 ± 234	*p* < 0.001	*p* = 1 (ZnMg1)

## Data Availability

The original contributions of this study are described in the article, and additional questions can be directed to the corresponding author.
